# Analysis of Aging Characteristics of Umbrella Skirts of Composite Insulators Operating under the Influence of a Wind and Sand Environment in South Xinjiang

**DOI:** 10.3390/ma17030680

**Published:** 2024-01-31

**Authors:** Xiaojun Zhang, Shilong Kuang, Suzhou Wu, Wenbing Zhuang, Chunqing He

**Affiliations:** 1 Xinjiang Electric Power Research Institute, Urumqi 830013, China; 2 Xinjiang Key Laboratory of Extreme Environment Operation and Detection Technology of Power Transmission & Transformation Equipment, Urumqi 830013, China; 3 School of Physics and Technology, Wuhan University, Wuhan 430072, China

**Keywords:** composite insulator, aging, southern Xinjiang area, wind and sand environment

## Abstract

To study the influence of multi-factors, such as long sunshine, sand erosion, and so on, in southern Xinjiang, we selected two kinds of composite insulators for the transmission lines in southern Xinjiang to study the aging characteristics of the umbrella skirt surface. The results of scanning electron microscope (SEM) and X-ray photoelectron spectroscopy (XPS) show that the surface roughness of the umbrella skirt is high and there are a large number of micron inorganic particles. The skirt has the characteristics of low C/O element ratio and high Al element content. The results of thermogravimetric analysis and micro infrared test show that the aging depth of the Myli Line skirt after 19 years of operation is 160~190 µm and that of Yuhe Line 1 after 14 years of operation is greater than 180 µm. The plasma discharge method was used to simulate the corona discharge in the actual operation to accelerate the aging of the surface of the umbrella skirt and the hydrophobic recovery of the umbrella skirt was investigated. The results show that the temperature has a great influence on the surface hydrophobic recovery performance after plasma treatment. These results may provide some theoretical guidance and technical support for the selection, operation, and maintenance of composite insulators in Xinjiang.

## 1. Introduction

Silicone rubber composite insulators have been widely used in high voltage transmission lines because of their good hydrophobic and water-repellent mobility, excellent anti-fouling flashover performance, low cost, light weight, and easy installation, etc. [1]. The main structure of the composite insulator is composed of a high-temperature vulcanized silicone rubber umbrella skirt and sheath, glass steel core rod, pressure balancing ring, and end fittings. Among them, silicone rubber umbrella skirts and sheaths are the key to ensuring that composite insulators have excellent external insulation performance. The main components of the silicone rubber umbrella skirt and sheath include silicone resin, aluminum hydroxide (ATH), white carbon black, etc., and, in addition, a small amount of other organic additives (such as vulcanizing agents, coupling agents, various silicone oils, etc.) and inorganic additives (zinc oxide, inorganic pigments, etc.). These component materials and formulations have an important effect on the quality and service life of silicone rubber umbrella skirt sheaths and even composite insulators. The topography of the southern Xinjiang region is dominated by the Gobi Desert, resulting in complex climatic and geological conditions and large temperature differences between day and night. Not only that, the transmission lines are always subjected to winds of Level 5 (speed of 8.0–10.7 m/s) and Local Level 7 (speed of 13.9–17.1 m/s) and some wind gusts even reach Level 8 to 9 (speed of 17.2–24.4 m/s), with the instant maximum wind up to Level 12 (speed of 32.7–36.9 m/s) or so, accompanied by a large amount of sand and dust [2].

Under the combined effect of environmental conditions, such as strong wind, sand, and large temperature difference, the surface of the composite insulator silicone rubber umbrella skirts will age, via chalking and erosion, which will make its surface structure rough and hydrophobic performance decline. Silicone rubber composite insulators are not only affected by natural conditions in the actual use process but also under the action of a high voltage electric field, which has a more serious effect on the aging of silicone rubber. The impact of charged particles generated by corona or arc discharge during the operation of silicone rubber, such as the impact of ions and electrons, causes the main chain of silicone rubber to break and cause molecular depolymerization. At the same time, the energy generated by the discharge causes the decomposition and reaction of gases, such as oxygen, in the surrounding air, producing highly oxidizing substances, such as active oxygen atoms, ozone, nitrogen oxides, etc., which can also cause the break of the molecular chain of silicone rubber and destroy the performance of silicone rubber. The role of corona arc can also cause local high temperatures and molecular chain destruction on the surface of silicone rubber, forming a conductive carbonization channel on the surface of silicone rubber, resulting in the decline of the mechanical and electrical properties of silicone rubber materials [3], and, ultimately, maybe harming the stable operation of the transmission lines [4].

In recent years, many research groups have carried out various research on silicone rubber materials. Shaik, M.G. et al. [5] developed an indigenous low-cost electrical tracking device for the tracking of fresh and aged polymer insulators and proposed new insights into the degradation mechanisms in terms of the electrical properties of polymer insulators and physicochemical changes in the material. Kaneko, T. et al. [6] tested the SiR tablets by using the terahertz (THz) absorption spectrum, infrared absorption spectrum, nuclear magnetic resonance, and indenter modulus, etc. According to aging conditions, they proposed three degradation mechanisms. Akbar, M. et al. [7] discussed the degradation of composite insulators in various outdoor working environments and the results of various studies on retarding the aging process by adding micron- and nano-scale organic filler particles to the base polymer materials were summarized. Sarathi, R. et al. [8] discussed the phenomenon of the leakage and marking of silicone rubber material with ammonium chloride as a pollutant under AC and DC voltage. It was concluded that surface aging is caused by degradation. Rahmat U.et al. [9] carried out multiple stress-accelerated aging experiments under AC and DC voltage. The service life of HTV-SR matrix composites was predicted under typical multiple-stress aging conditions. Chandrasekar, S. et al. [10] analyzed the influence of applied voltage, conductivity, and contaminant velocity on electric trace. The trend of leakage current was analyzed by the moving average technique. Faiza et al. enhanced the structural stability and hydrophobic behavior of silicone rubber by adding nano-composite fillers to the polymer chain. Tahir, M.H. et al. [11] studied the hydrophobicity loss and recovery of four kinds of silicone rubber micro-nano composite materials under corona discharge. They found that the sample recovery time was proportional to the duration of exposure to the corona discharge and inversely proportional to the electrode-to-sample gap. Esmaieli, M. et al. [12] carried out a harmonic analysis of the leakage current signal. The experiments of the asymmetric aging, complete aging, and original insulator were carried out to analyze the condition of the composite insulator. Thipprasert, W.et al. [13] studied electrical performance and deterioration at the insulator’s surface under the wheel test conditions. Birtwhistle, D. et al. [14] carried out a study of the aging insulator using X-ray photoelectron spectroscopy (XPS) measurements and found that the aging rubber surface aluminum content increases and silicon content decreases. Hillborg, H. et al. [15] found that cyclic siloxane small molecules are generated from silicone rubber during corona discharge and confirmed that these small molecules are involved in the hydrophobicity restoration process.

There are many tests and studies of composite insulators for different environments. However, few people have analyzed the aging characteristics of actual samples in harsh environments, such as extreme temperatures and wind-sand in Xinjiang. In this paper, the surface of composite insulator umbrella skirts in operation on two transmission lines of the Xinjiang Power Grid will be selected for surface morphology and elemental characterization, aging depth tests, and hydrophobic recovery tests, aiming to study the aging characteristics of composite insulators in operation under the sandy and windy environments in the southern Xinjiang region and analyze the causes of aging. This paper can provide some theoretical guidance and technical support for the selection operation and maintenance of composite insulators in the southern Xinjiang area of the State Grid.

## 2. Experimental

### 2.1. Sample Information

**Figure 1:**
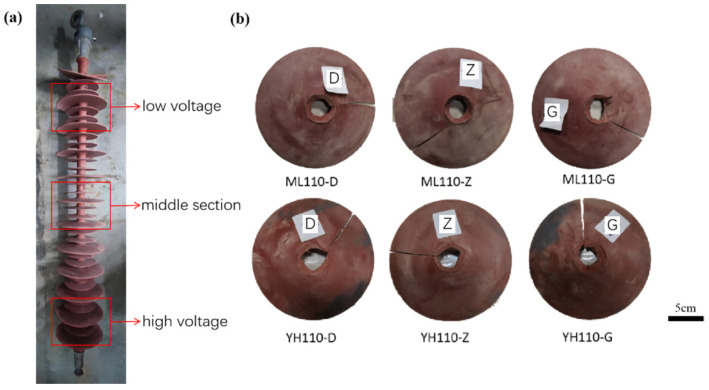
Sampling diagram (**a**) and appearance of umbrella skirt samples (**b**).

The samples used in this paper are the composite insulators of the 110 kV Myli Line and 110 kV Yuhe Line in the South Xinjiang area; the samples are taken from different positions of the umbrella skirts for testing, as shown in Table 1. The appearance of the samples is shown in Figure 1. The specific meaning of pollution level is that in areas with moderate atmospheric pollution, the salt density on the surface of the umbrella skirt is 0.06–0.1 mg/cm^3^.

### 2.2. Surface Topography and Elemental Characterization and Test Methods

Surface Microscopic Morphology Test: Small cubes with dimensions of 5 mm × 5 mm × 2 mm were cut on the surface of the umbrella skirt samples as specimens. Before the test, the surface of the sample was sprayed with gold; the processing time was 100 s. A scanning electron microscope (SEM) of S-4800 (Hitachi, Wuhan, China) was used to characterize the surface micro-morphology of the umbrella skirt samples; the working voltage of the instrument was 10 kV.

Surface Elemental Content Test: Small cubes with dimensions of 10 mm × 5 mm × 2 mm were cut on the surface of the umbrella skirt samples as specimens. The VG Multilab 2000 X-ray Photoelectron Spectrometer (XPS) (Thermo Fisher Scientific, Wuhan, China) was used to characterize the relative elemental content of the surface of the umbrella skirt samples, with a test voltage of 12 kV and a full-spectrum energy scanning range of 0~1350 eV, with a step size of 0.5 eV. Sample specifications were the same as those of the surface micromorphology test. The analyzed data were calibrated against a C1s binding energy of 284.8 eV.

### 2.3. Surface Aging Depth Test and Test Method

Thermogravimetric Test: We cut a small piece of about 0.6 cm × 0.4 cm in the area to be measured in the umbrella skirt and then used a Leica CM1850 frozen microslicer (Leica Biosystems Nussloch Gmbh, Wuhan, China) to slice the sample piece layer by layer, starting from the upper surface. The specific slicing steps were as follows: First, we smoothed the lower surface. Then, the smoothed lower surface was fixed on the sample table of the microtome with adhesive and the slice thickness was set to 30 µm. The cold table was started to cool the sample before slicing. The slices of the silicone rubber umbrella skirt were collected successively and every two slices (i.e., samples with a depth of 60 µm) were analyzed in a group for TG-DSC testing. Two consecutive slices of the cut ultrathin slice samples were taken as a group and four groups of samples were prepared. A TGA2/DSC3 thermogravimetric analyzer (Mettler Toledo, Wuhan, China) was used to test the mass change of the samples during the warming process, with a warming rate of 20 °C/min and a temperature range of 30–800 °C.

Depth of Aging Test: Frozen slices were cut perpendicular to the surface of the umbrella skirts and, due to the characteristics of the samples, the thickness of the slices varied from 100 to 200 µm and the ultra-thin slices were required to contain the complete upper surface. The sliced samples were scanned using the reflectance mode of a Nicolet In10 Fourier Transform Microinfrared Spectrometer (FTIR) (Thermo Fisher Scientific, Shanghai, China). Surface scanning refers to the measurement of the diaphragm moving in a selected plane area; the brightness of the spot and the size of the diaphragm are scanned in the same line during the test, the scanning area is 200–150 µm, and the step length is 5 µm.

### 2.4. Surface Hydrophobic Recovery Test

Plasma Treatment: We took the more serious degree of pollution on the sample sector as a specimen, using the modified YZD08-5C plasma cleaner (Sat Plasma, Wuhan, China) to accelerate the aging of the sample surface treatment to simulate the actual operation of the corona discharge phenomenon. Plasma discharge treatment conditions [16,17]: the working gas was argon, the gas flow rate was controlled at 200–300 mL/min, the voltage in the reaction bin was about 4.0 Pa during the glow discharge, the discharge power was 40 W, and the discharge time was 3 min.

Hydrophobicity Test: We set the relative humidity of the B-T-107D constant temperature and humidity box to 35%; set the temperature to −35 °C, 0 °C, and −35 °C, respectively; and put the plasma-treated specimens into the constant temperature and humidity box to recover for 48 h. Because the consistency between the HC grading method (water spray grading method) and the static contact angle method among the hydrophobicity test methods was poor [18,19], the hydrophobicity of the umbrella skirt as a whole, measured by the HC grading method, could more comprehensively reflect the aging of umbrella skirts surface compared with the static contact angle test; therefore, this study adopted the HC grading method to analyze the hydrophobicity of umbrella skirts. 

Analysis of Siloxane Small Molecules: About 5 g of particles were cut on the surface of the insulator umbrella skirt samples as a specimen and the extract was heated by a Soxhlet extractor using n-hexane as a solution at 90 °C for 96 h to obtain the extract liquid solution, which was concentrated to 10 mL and then analyzed by chromatography and mass spectrometry using 450GC-320MS gas-chromatography–mass-spectrometry (GC–MS) (Varian, Wuhan, China). The gas chromatography was performed on a VF-5 ms column with the inlet temperature set to 300 °C; the column was first set to 50 °C for 3 min and then ramped up to 290 °C at a temperature increase rate of 20 °C/min for 10 min. High-purity helium (≥99.999%) was used as the carrier gas at a flow rate of 1 mL/min and the sample was injected into the sample with a non-split flow in a volume of 1 µL. The EI mode was chosen for mass spectrometry and the ionization energy was set to 1 µL. The EI mode was selected for the mass spectrometry, with an ionization energy of 70 eV and a mass scan range of (30–2000) *m*/*z* (AMU). Due to the characteristics of the instrument, only small siloxane molecules with a cyclic unit of 20 could be detected.

## 3. Results and Discussion

### 3.1. Surface Morphology and Surface Element Analysis

**Figure 2:**
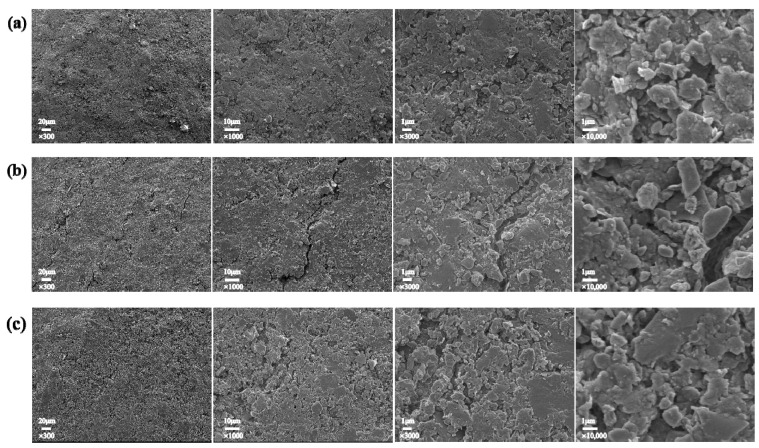
Surface topography of ML110-D (**a**), ML110-Z (**b**), and ML110-G (**c**).

**Figure 3:**
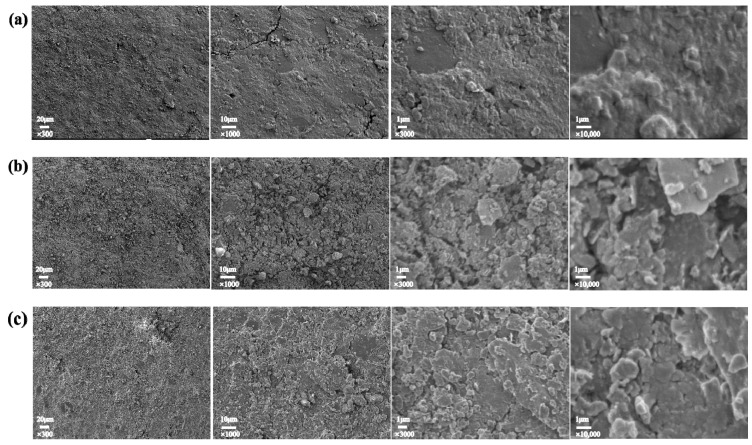
Surface topography of YH110-D (**a**), YH110-Z (**b**), and YH110-G (**c**).

The surface morphologies of the umbrella skirts at the low voltage, the middle section, and the high voltage of the 110 kV Myli Line and the 110 kV Yuhe Line in the South Xinjiang region are shown in Figure 2 and Figure 3. From left to right, the surface morphologies of umbrella skirts are 300 times, 1000 times, 3000 times, and 10,000 times, respectively.

The literature [20] shows that the surface of the unaged silicone rubber umbrella skirts of composite insulators is a dense, defect-free, and continuous surface. According to the SEM results, a large number of unevenly distributed micron-sized inorganic particles exist on the surface of the aging umbrella skirts under high-temperature wind and sand environments and the surface is relatively rough.

The depth of analysis of the XPS test is within 10 nm of the surface, which can reflect the chemical composition state of the nanometer scale on the surface of silicone rubber umbrella skirts [21,22]. The relative contents of the elements on the surfaces of the 110 kV Myli Line and 110 kV Yuhe one Line umbrella skirts are as shown Table 2.

**Figure 4:**
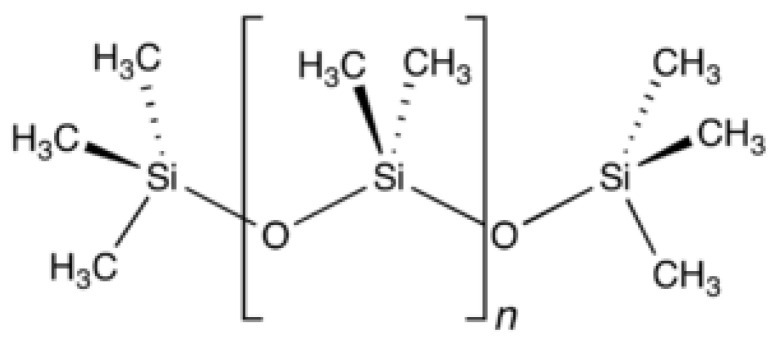
Molecular structure of PDMS.

The structure of PDMS (polydimethylsiloxane) [23], the main organic component of the umbrella skirts, is shown in Figure 4. According to the molecular structure, it can be calculated that the ratio of its three elements, C/O/Si, is 2:1:1 while SiO_2_ and ATH, as inorganic fillers, are dispersed in the matrix and surrounded by PDMS so that the ratio of the three elements, C, O, and Si, on the surface of the silicone rubber is about 2:1:1 in the absence of the aging of the organic component.

When combined with Table 2, it can be seen that the relative content of the C element is low in both groups of samples, the relative content ratio with the Si element is less than 2:1, there is side-chain methyl decomposition, the surface begins to become inorganic, and the surface C/O ratio is lower than the inner layer, indicating that there is a phenomenon of aging on the surface; the ratio of n(C)/n(O) in the Yuhe Line and the first line is even smaller, which indicates that the surface aging is relatively more serious. The higher relative content of Al elements indicates that the aluminum hydroxide wrapped by PDMS is beginning to be exposed and, combined with the SEM surface morphology analysis, it is inferred that the umbrella skirt silicone rubber has been oxidized and decomposed with cracks on the surface.

In the Introduction, we mentioned that composite insulators in Xinjiang have long operated in high-temperature and windy sand areas. It can be seen from the XPS results that the Methyl degradation of the side chain of PDMS on the surface of the sample is serious. The Si–O–Si main chain of the PDMS is destroyed under the action of high temperatures and oxygen. At the same time, a side group oxidation reaction occurs, which degrades the macromolecules on the surface of silicone rubber and reduces the molecular weight. In addition, when the PDMS molecular chain breaks, it is accompanied by the generation of free radicals, e.g., silicon–carbon bonds and carbon–hydrogen bonds will break to form -Si, -CH3, and -H [6]. During the thermal aging process of the silicone rubber surface, the internal filler decomposes and is exposed to the silicone rubber surface. These free radicals cross-link with adjacent chemical bonds, greatly reducing the flexibility of silicone rubber macromolecular chains, and the surface of the material becomes hard and brittle. In the windy sand environment, the phenomenon of cracking and pulverization easily appears in the SEM results. We can see that a large number of micron-level inorganic particles are distributed on the surface of the sample and the surface roughness of the material is relatively high. This is because during the thermal aging process of HTV, the internal ATH decomposes into aluminum oxide and water and the Si–OH on the surface of white carbon black undergos a condensation reaction to generate inorganic particles of SiO_2_ and water, which are exposed to the surface of the material, increasing the surface roughness; with the extension of the thermal aging time, the filler particles on the surface of the material also increase.

### 3.2. Aging In-Depth Analysis

**Figure 5:**
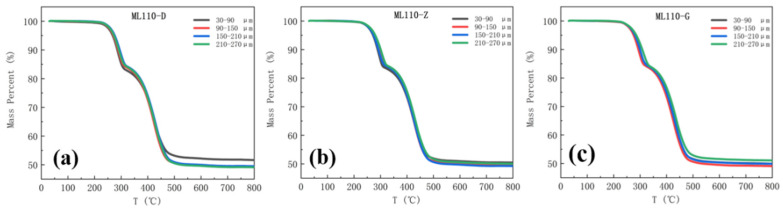
Thermal weight loss plots of umbrella skirts at different positions of ML110-D (**a**), ML110-Z (**b**), and ML110-G (**c**).

**Figure 6:**
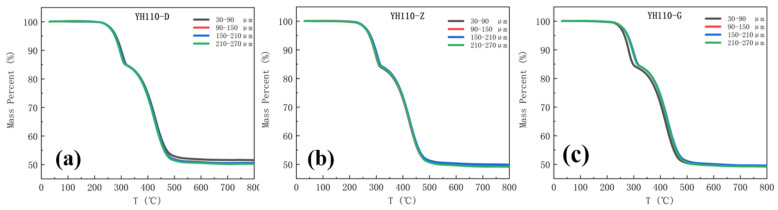
Thermal weight loss plots of umbrella skirts at different positions of YH110-D (**a**), YH110-Z (**b**), and YH110-G (**c**).

The black, red, blue, and green curves in the heat loss diagram, respectively, show the thermal decomposition of the samples in the depth ranges of 30–90 µm, 90–150 µm, 150–210 µm, and 210–270 µm from the surface. The 110 kV Myli Line and 110 kV Yuhe Line 1 heat loss curves are shown in Figure 5 and Figure 6.

**Figure 7:**
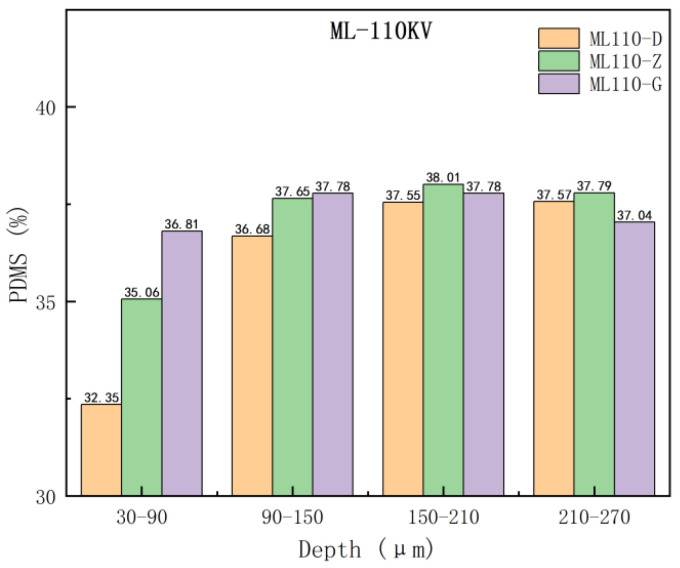
PDMS content of umbrella skirt samples at different locations of the Myli Line.

**Figure 8:**
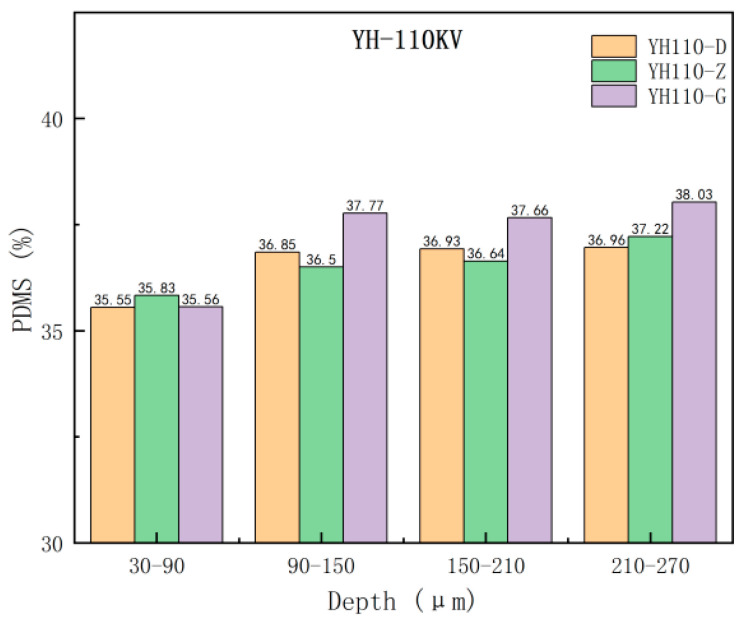
PDMS content of umbrella skirt samples at different locations of the Yuhe Line.

From Figure 5 and Figure 6, it can be seen that the trend of the thermal decomposition of each group of samples of the same umbrella skirts is approximately the same; the curves are highly overlapped in the pre-stage of the thermal decomposition and the aging analysis of the surface of the composite insulator umbrella skirts in the southern Xinjiang region shows some differences in the later stage. To further investigate the PDMS component content of the samples with different depths, the thermal weight loss rate of each sample between 30–320 °C and 320–700 °C was recorded and the change of the PDMS component content of each sample was obtained by formula calculation. The heat weight loss rate and the calculated PDMS content of each sample are shown in Figure 7 and Figure 8.

The PDMS content of the samples in different umbrella skirts was plotted in the same picture to observe the changes; the purple, green, and orange data columns represent the PDMS content of the samples in the umbrella skirts at the high voltage, the middle section, and the low voltage, as shown in Figure 7 and Figure 8.

In Analysis Table 3 and Table 4, it can be seen that as the depth of each umbrella skirt sample increases, the content of its PDMS component also increases and gradually tends toward stabilization. The PDMS contents of the aging part of the umbrella skirts are relatively low and the content of the unaged part is relatively high, from which the aging depth of the umbrella skirts can be roughly judged. The different environments of each line will lead to the difference in the PDMS content of each line. From Figure 7 and Figure 8, we can determine that the aging depths of ML110-D, ML110-Z, and ML110-G are all within 150–210 µm; the aging depths of YH110-D, YH110-Z, and YH110-G are all within 150–210 µm.

The micro-infrared results can visualize the aging depth of the umbrella skirts; the area distribution of the methyl peaks at wave number 2960 cm^−1^ is shown below. The blue color indicates that the absorption of the methyl peak is very low, indicating that the content of PDMS is very low; the red color indicates that the absorption of the methyl peak is high, indicating that the content of PDMS is high; and the area between the two is the aging area of the sample.

**Figure 9:**
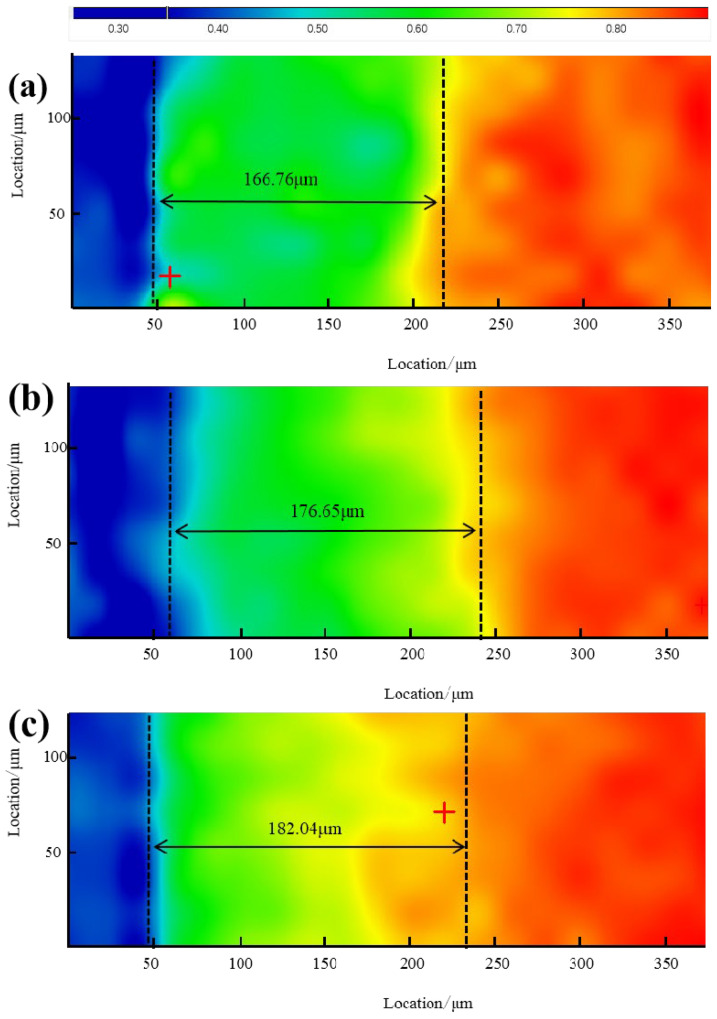
Schematic representation of the aging depth of different umbrella skirt samples of ML110-D (**a**), ML110-Z (**b**), and ML110-G (**c**).

**Figure 10:**
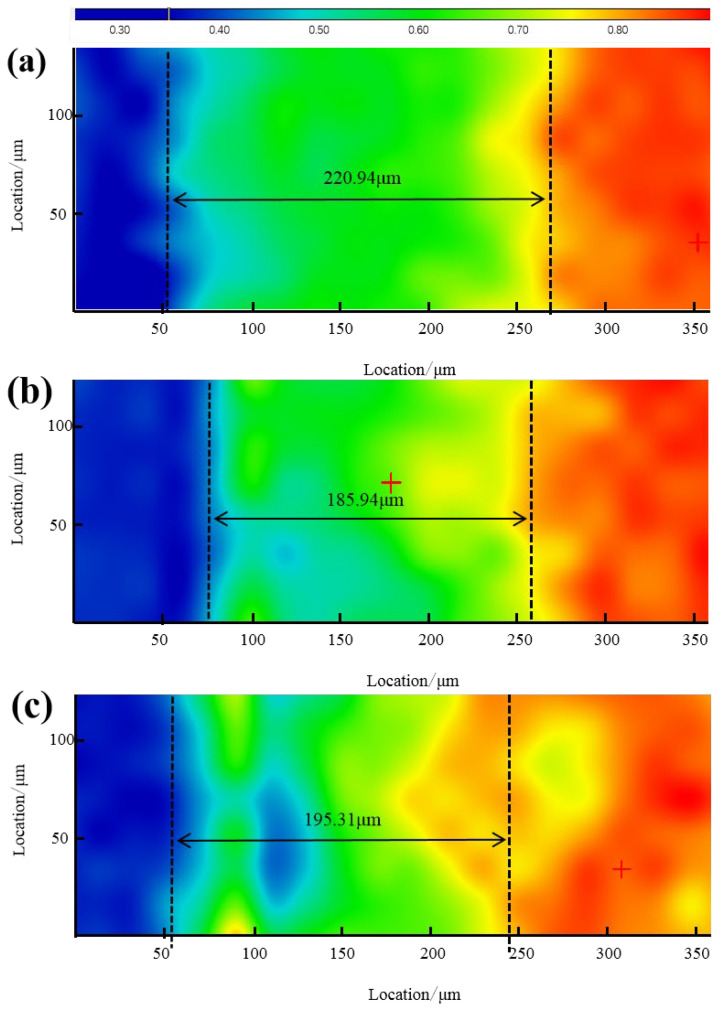
Schematic diagram of the aging depth of different umbrella skirt samples of YH110-D (**a**), YH110-Z (**b**), and YH110-G (**c**).

As can be seen from Figure 9 and Figure 10, the aging depth of the skirt is 182.04 µm at the high voltage, >176.65 µm at the middle section, and >166.76 µm at the low voltage. The aging depth of the Yuhe Line umbrella skirt is 220.94 µm in low voltage, >195.31 µm in high voltage, and >185.94 µm in the middle section; the aging depth of the Myli Line is generally lower than that of the Yuhe Line.

It can be seen from the micro-infrared results that the methyl content of the aging part on the surface of the composite insulator umbrella is reduced. In this case, the aging depth of the skirt surface can be well characterized by the distribution of methyl concentration. Thermogravimetric experiments show that the PDMS content increases with the increase in test depth. The results show that the surface microstructure of the silicone rubber umbrella skirt changes significantly due to aging. On the one hand, the main Si–O bond is broken and the side chain methyl is oxidized. On the other hand, the aging only occurs on the surface and the deep layer of the silicone rubber matrix is not affected too much. However, according to the combined micro-infrared and thermogravimetric results, its aging does not show an obvious law and the aging of the Yuhe Line with a short operating life is more serious than that of the Myli Line with a long operating life, reflecting certain irregular aging characteristics. We speculate that the main reason is that under the influence of strong winds in Xinjiang, large particles of sand and dust in the air will have a certain etching and weathering effect on the surface of the umbrella skirt. The specific influencing factors, such as wind direction and windward slope, may need further experiments to study.

### 3.3. Hydrophobic Restoration Studies

The main chain of PDMS is hydrophilic Si–O–Si and the side chain is hydrophobic-CH3, which has a shielding effect on the main chain, making the surface of the umbrella skirts hydrophobic [24]. After aging, the hydrophobic groups, such as the side chain -CH3, are reduced or the orientation of the groups is shifted to the matrix and the hydrophilic groups are exposed, making the surface of the umbrella skirts hydrophilic [15]. In this paper, we simulate the actual operating temperature environment in the South Xinjiang region and study the surface hydrophobicity recovery of the umbrella skirts at three different temperatures: +35 °C, 0 °C, and −35 °C.

**Figure 11:**
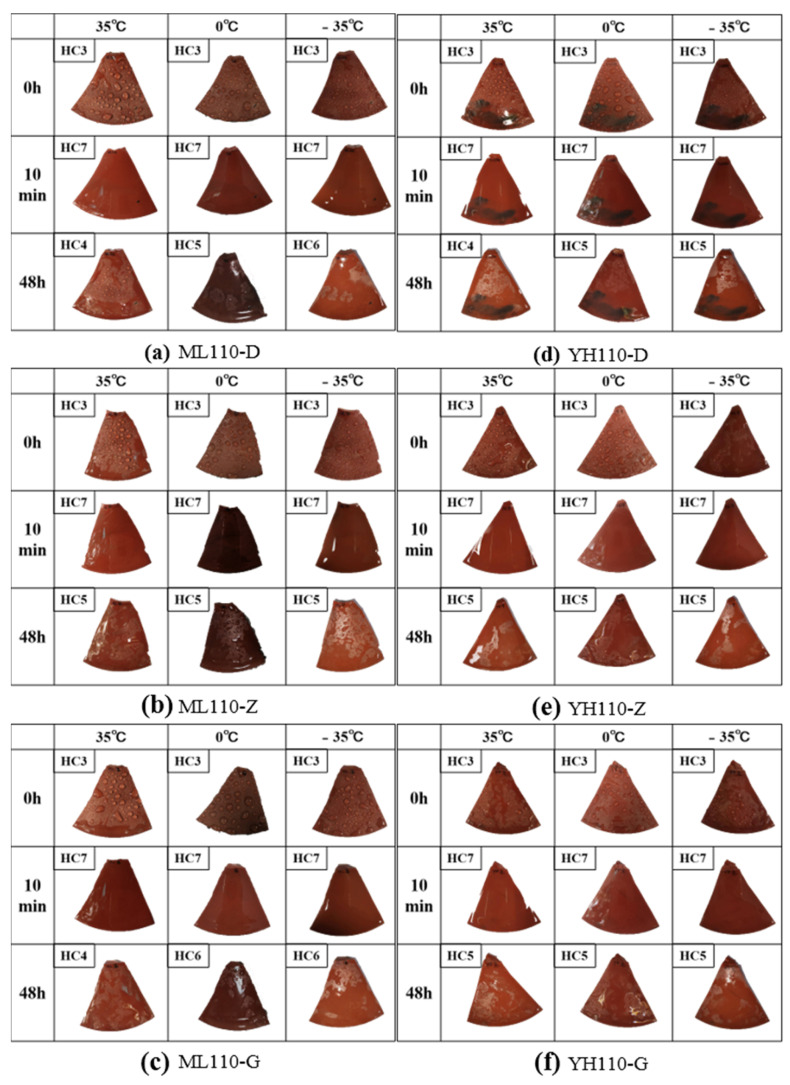
Recovery of the hydrophobicity of ML110-D (**a**), ML110-Z (**b**), ML110-G (**c**), YH110-D (**d**), YH110-Z (**e**), and YH110-G (**f**) at different temperatures.

The 110 kV Myli Line and 110 kV Yuhe Line umbrella skirt specimens are analyzed at three different temperatures: before plasma discharge treatment, 10 min after treatment, and 48 h after treatment to conduct a water spray grading test; the results are shown in Figure 11.

At the end of the plasma treatment, the surface of all composite insulators changed from a hydrophobic state to a hydrophilic state; at this time, the hydrophobicity of the umbrella skirts surface grading was grade HC7. Under the action of intramolecular stress, the inorganic dense layer ruptured and produced cracks and small molecules in the silicone rubber body diffused to the surface along these cracks, making the sample hydrophobicity gradually recover [25]. From the Figure 11 grading results, it can be seen that the best hydrophobic recovery effect is achieved at +35 °C while the hydrophobic recovery effect is poor at 0 °C and −35 °C.

**Figure 12:**
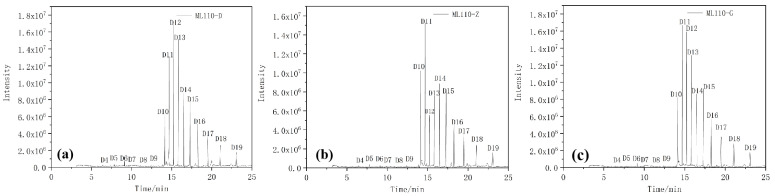
Chromatograms of different umbrella skirt samples of ML110-D (**a**), ML110-Z (**b**), and ML110-G (**c**).

**Figure 13:**
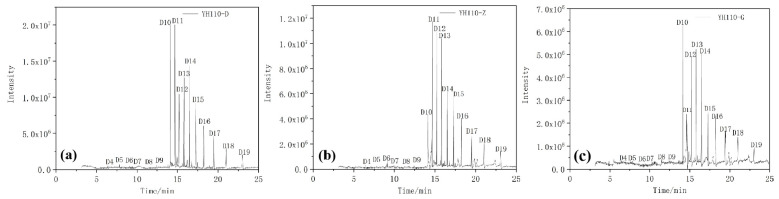
Chromatograms of different umbrella skirt samples of YH110-D (**a**), YH110-Z (**b**), and YH110-G (**c**).

The chromatograms of the extracts analyzed by GC–MS are shown in Figure 12 and Figure 13.

The hydrophobic recovery of composite insulators can be used to make a macroscopic judgment on the aging degree of composite insulators; the hydrophobic recovery is closely related to the types and content of silane small molecules contained in the silicone rubber of composite insulators [26]. In the previous discussion, we found that aging will make the flat and smooth surface of silicone rubber rough and produce physical defects, such as holes and cracks. This will cause the hydrophobicity and electrical insulation of silicone rubber to be broken, shorten the life of silicone rubber, and gradually reduce its use value. The surface of silicone rubber becomes hard and brittle and the internal filler is exposed, which reduces the hydrophobicity and insulation of its surface. Through the data of GC–MS, we found that after long-term operation, the small molecules of D9 and below on the silicone rubber surface are gradually consumed and the small molecules of D10 and above remain relatively more as they can exist more stably in composite insulators. Combined with the SEM image results, the aging process also produces micropores and surface cracks, which provide a channel for the small molecules of siloxane in the internal silicone rubber to diffuse to the surface. It makes the surface of the skirt sample maintain hydrophobic restorative properties after over 10 years of operation. However, although the surface of the aging insulator parachute skirt still has certain hydrophobic restorative properties, its hydrophobic performance has declined, resulting in the decline of mechanical and electrical properties of silicone rubber materials.

## 4. Conclusions

(1) The umbrella skirts operating in the southern border area have been affected by ultraviolet rays, wind, sand, and other aging factors for a long time, which makes the surface of the umbrella skirts chalk and crack. The surface layer of PDMS has been oxidized and decomposed so that the inorganic filler is exposed on the surface to form a large number of inorganic particles and become rough;

(2) Insulator running time and operating environment have an influence on the aging state of the umbrella skirt surface. The aging depth of the Myli Line umbrella skirts with 19 years of operation is between 160 and 190 µm while the aging depth of the Yuhe Line umbrella skirts with 14 years of operation is between 180 and 220 µm. The surface aging of the insulator umbrella skirts of the two lines is deeper and the aging of the Yuhe Line with shorter operation times is more serious. It can be seen that the erosion of the insulator is more serious in the wind and sand environment in the Yuhe Line area;

(3) The hydrophobic grades of the analyzed samples are between HC3 and HC4 and the hydrophobic properties have decreased. The effect of operating years on the recovery of water repellency on the surface of umbrella skirts is not obvious and the water repellency of umbrella skirts recovers better under high-temperature conditions; however, the water repellency recovery performance is poorer at low temperatures;

(4) At present, there are two mitigation strategies for the aging of composite insulator umbrella skirts in the Xinjiang region. The first one is that due to the high temperature in the Xinjiang region, heat-resistant fillers or additives can be added to make silicone rubber have better heat resistance. Second, since the aging of silicone rubber only occurs on the surface in most cases, and its interior is not damaged, a layer of repair material is sprayed on the surface, which can effectively alleviate the surface aging.

## Figures and Tables

**Table 1:** The sample number and the operating environment information of insulators.

Line	Sample Number	Voltage Level	Umbrella Skirt Position	Dirtiness	Operating Period/Year	Highest Temperature/°C	Operating Environment
Myli	ML110-D	110 kV	low voltage	c	19	45	Wind, sand, heat
	ML110-Z		middle section				
	ML110-G		high voltage				
Yuhe	YH110-D	110 kV	low voltage	c	14	42	Wind, sand, heat
	YH110-Z		middle section				
	YH110-G		high voltage				

**Table 2:** Relative content of elements on the surface of the samples.

Sample Number	Elemental Content/%				n(C)/n(O)/n(Si)	n(C)/n(O)
	C	O	Si	Al		
ML110-D	47.14	28.15	20.97	3.75	2.24:1.34:1	1.67
ML110-Z	43.07	29.52	22.43	4.99	1.92:1.32:1	1.46
ML110-G	43.18	29.33	22.97	4.53	1.88:1.28:1	1.47
YH110-D	38.08	31.33	24.84	5.04	1.53:1.26:1	1.22
YH110-Z	40.85	29.37	23.73	4.80	1.72:1.23:1	1.39
YH110-G	45.44	26.99	23.56	3.67	1.93:1.14:1	1.68

**Table 3:** Thermal weight loss rate and relative content of organic fractions in Myli Line umbrella skirt samples.

Samples	Mass Loss/% (30–320 °C)	Mass Loss/% (320–700 °C)	Relative PDMS Content/%
ML110-D1	19.96%	28.23%	32.35%
ML110-D2	19.24%	31.42%	36.68%
ML110-D3	18.48%	31.97%	37.55%
ML110-D4	18.79%	32.03%	37.57%
ML110-Z1	19.27%	30.19%	35.06%
ML110-Z2	18.40%	32.03%	37.65%
ML110-Z3	18.43%	32.31%	38.01%
ML110-Z4	17.78%	32.04%	37.79%
ML110-G1	18.48%	31.40%	36.81%
ML110-G2	18.63%	32.17%	37.78%
ML110-G3	17.94%	32.06%	37.78%
ML110-G4	17.38%	31.41%	37.04%

**Table 4:** Thermal weight loss rate and relative content of organic fractions of Yuhe Line umbrella skirt samples.

Samples	Mass Loss/% (30–320 °C)	Mass Loss/% (320–700 °C)	Relative PDMS Content/%
YH110-D1	15.15%	33.35%	40.04%
YH110-D2	15.09%	34.33%	41.34%
YH110-D3	15.21%	34.40%	41.40%
YH110-D4	15.92%	34.59%	41.51%
YH110-Z1	16.22%	33.91%	40.56%
YH110-Z2	15.91%	34.48%	41.37%
YH110-Z3	15.66%	34.35%	41.25%
YH110-Z4	15.92%	34.88%	41.89%
YH110-G1	16.53%	34.11%	40.75%
YH110-G2	15.56%	35.02%	42.14%
YH110-G3	15.46%	34.89%	41.99%
YH110-G4	15.49%	35.22%	42.42%

## Data Availability

Raw data are available upon request.
